# Plant-Based Only: Investigating Consumers’ Sensory Perception, Motivation, and Knowledge of Different Plant-Based Alternative Products on the Market

**DOI:** 10.3390/foods11152339

**Published:** 2022-08-05

**Authors:** Marcel Pointke, Marlene Ohlau, Antje Risius, Elke Pawelzik

**Affiliations:** 1 Division of Quality of Plant Products, Department of Crop Sciences, University of Goettingen, 37075 Goettingen, Germany; 2 Marketing for Food and Agricultural Products, Department of Agricultural Economics and Rural Development, University of Goettingen, 37073 Goettingen, Germany

**Keywords:** cheese alternative products, meat alternative products, milk alternative products, consumer research, consumer profiling, sensory characterization, RATA

## Abstract

Consumer acceptance and product development of sustainable, healthy, and tasty plant-based alternative products (PBAPs) are closely interlinked. However, information on consumer perceptions of the sensory profile of plant-based meat, cheese, and milk remains scarce. The study aimed to investigate German consumers’ (1) sensory evaluation of PBAPs and (2) consumers´ motivations and knowledge underlying the purchase of such products. This was analyzed in relation to different dietary styles of consumers (omnivore, flexitarian, vegetarian, vegan). A sample of 159 adults completed two tasks: first, a sensory test in which participants tasted and rated three different PBAPs in two consecutive sessions, and second, a questionnaire on consumption behavior, motivation, and knowledge. Results show few differences between nutrition styles in sensory evaluation of individual product attributes. However, overall liking was rated significantly higher by vegans than by omnivores. All dietary styles reported animal welfare and environmental aspects as the main motivations for consuming PBAPs. Most participants acknowledged that meat and cheese alternatives are highly processed foods and not a fad but are not automatically healthier or more environmentally friendly than their animal-based counterparts. Future research should focus on emerging product segments such as plant-based cheeses to better understand how consumers evaluate PBAPs.

## 1. Introduction

Reducing the global consumption of animal-based products and changing to a predominantly plant-based diet are necessary approaches for a sustainable diet that simultaneously has positive environmental and human health and public health impacts [1]. An unbalanced diet low in fruits, vegetables, nuts, and whole grains, high in red and processed meats, with a high sodium intake, is responsible for the greatest health burden worldwide [2]. The EAT Lancet Commission’s published planetary health diet states that meat and dairy constitute important parts of the diet but in significantly smaller proportions than plant-based foods [3]. Additionally, according to the recommendations of the German Nutrition Society, a reduction in meat consumption in particular is necessary. With a per capita consumption of 57.3 kg for the year 2020 [4], meat consumption is still significantly above the recommended consumption levels, requiring many people in the Western world to make far-reaching changes to their eating habits. Meat has long been an integral part of traditional German meals and, for many, an important part of their food culture. Although there has been increasing interest in reducing meat consumption in Germany, many social and personal barriers prevent this dietary change, as meat is often perceived as a status symbol [5]. Other reasons are taste preferences, enjoyment and habit [6,7], the social environment, or a lack of awareness of the link between climate change and food consumption [7,8,9]. A 2020 online survey of German young adults showed that just under 13% abstained from meat, which is about twice as many as in the overall German population [10]. Spiller et al. [10] showed that giving up meat was a trend among the survey respondents, as around one-third of those who ate a vegetarian or vegan diet had switched to a meat-free diet only a year earlier.

In making a dietary change to reduce animal-based product consumption, plant-based alternative products (PBAPs) can make an important contribution to managing those changes within the familiar structure of meals and existing cooking skills, but also increase product variety as well as availability, which can otherwise be considered barriers to dietary change [11,12,13]. PBAPs are products composed of predominantly plant-based proteins, such as legumes, grains, nuts, seeds, mushrooms, and fats. They have slowly but steadily increased their market share in Germany, with many supermarkets and discount stores increasing the range of available products [14]. The goal of alternative products is to mimic the animal-based product as closely as possible, so the sensory evaluation effort often aims to assess the similarities between these products and their traditional animal-based product. Consumer studies are necessary to evaluate the sensory characteristics and the perception during consumption in order to achieve sensory acceptability. However, there are currently limited data on which sensory aspects are important to consumers, particularly in the area of plant-based cheese alternatives and the most commonly used substitutions. Process optimization and new technologies to utilize and improve novel plant proteins are driving product development of PBAPs, but at the same time require constant evaluation of sensory properties from a consumer perspective.

While sensory research is an important tool for analyzing consumer preference and acceptance, it is also insufficient because sensory acceptance is only one factor that influences food choice and consumption [15]. Among various key determinants in the decision-making process, cognitive factors are considered critical to sustainable diets, as the shift to more sustainable lifestyles must take into account not only consumers’ individual needs and preferences but also their attitudes and knowledge about health and environmental values [16]. Studies suggest that PBAPs are more often perceived as being environmentally friendlier and, partly, healthier than their animal counterparts [17,18,19,20]. However, there is little research on what consumers actually know about the sustainability of PBAPs. In terms of environmental aspects, alternative products are likely less detrimental to the environment than most meat production due to refinement losses within the animal production line [21,22]. However, as PBAPs are often ultra-processed foods, the extensive processing required uses energy and resources and leads to losses during the transformation from the raw material into final products [22,23]. From a nutritional perspective, reduced consumption of red and processed meat and partial replacement of the original amount of meat with plant-based meat alternatives has been shown to have a beneficial effect on the intake of unsaturated fatty acids and dietary fiber [24,25]. Moreover, PBAPs represent an important source of vegetable protein [26]. Yet, these new food matrices are created from raw materials by adding other ingredients through food technology processes, so PBAPs often contain high levels of salt, sugar, and saturated fat, as well as flavorings and other additives [27,28,29]. Consumers have limited access to reliable scientific publications or the ability to evaluate robust scientific data. Their knowledge of sustainability-related properties of PBAPs depends on claims made by manufacturers and internet searches that generally do not provide clear, validated evidence for specific features. Several studies point out that relevant knowledge is a prerequisite to enabling consumers to make environmentally friendly choices [14,30,31]. Thus, it is important to better understand consumer knowledge related to sustainability-related attributes (e.g., environmental impact, health, degree of processing) of PBAPs.

This paper also attempted to determine whether consumers’ knowledge and motivation influence sensory evaluation. Some studies have shown that extrinsic factors, such as packaging, brand, information, emotion, and social environment, influence sensory perception [32,33,34]. Information focused on sustainability can especially influence consumers’ liking of food, as shown with various commodities, such as chocolate, coffee, and lamb [35], but has so far been little studied in relation to PBAPs. We intended to investigate whether information about the sustainability of PBAPs—which is conveyed along with consumer knowledge and motivation—acts as a cognitive impulse, thereby inducing a change in sensory perception. The research project in which this study was conducted focused on comparing different nutrition styles from a comprehensive sustainability perspective. For this reason, the participants were grouped according to their dietary preferences (omnivore, flexitarian, vegetarian, and vegan), and the data were analyzed accordingly. The primary focus was to determine the prevalence and acceptance of different plant-based foods in German diets. This research project investigated the existing range of PBAPs from three different product groups (plant-based salami cold cut, plant-based cheese, and plant-based oat drink) in food retail from a consumer perspective regarding availability and sensory quality. An assessment of PBAPs may be helpful for further development in product research and thus for a better understanding of the acceptance of such products. Specifically, the following questions arise: (i) How do consumers evaluate the sensory quality of products on the market, and does information on sustainability characteristics influence the sensory perception? (ii) What is the consumption pattern for these products? (iii) What knowledge and (iv) what motivations support the current products offered? These questions will be analyzed by comparing different dietary groups (omnivores, flexitarians, vegetarians, vegans).

## 2. Materials and Methods

### 2.1. Procedure

The study was conducted in January 2020 in the sensory laboratory of the Georg-August University of Goettingen. During the evaluation, each participant sat in a separate booth designed according to the specifications of ISO 8589 [36]. This study received ethical approval from the Ethics Committee of the University of Goettingen. All participants received an information letter stating the conditions for participation in the study and signed an informed consent form. Each participant received a EUR 5 expense allowance for their participation in a session of approximately 60 min. After receiving a short welcome and introductory information, the participants were seated in the booths. The products were tasted twice, in two successive sessions, with a 15 min break between sessions. During this break, sociodemographic variables (age, gender, education, and household income), frequency of, and motivation for, the consumption of plant-based alternative products, and knowledge of the sustainability features of such foods were obtained by questionnaire. This questionnaire served as a way to measure the influence of information on sensory perception. Thus, the questionnaire was intended to reveal a possible influence on the sensory evaluation of the second session. Data recording was performed with the software EyeQuestion^®^ (version 4.11.57, EyeQuestion^®^, Elst, The Netherlands) under white lighting, controlled temperature (20 ± 1 °C), and airflow conditions.

### 2.2. Sample Recruitment

Participants were recruited via notices posted at the University of Goettingen and via various social media channels. A sample size of 160 people in total participated; however, complete data from the sensory testing and survey were only received from 159 participants and included in the analysis (Table 1).

### 2.3. Description of the Plant-Based Alternative Products

Participants sampled three different plant-based product groups (milk, cheese, and salami). These were purchased from the local supermarket, and each had only minor variations in the best-before date of one week or less, so it can be assumed that no change in the formulation had occurred.

The plant-based milk alternative (PBMiA) was an oat drink from the company Alpro with the caption “Oats; purely vegetable, no added sugar” (Alpro GmbH, Duesseldorf, Germany); Alpro is the European market leader for soy-based foods and dominates the German food market [37].

SimplyV Natur, “Vegane Genießerscheiben,” a non-dairy sliced cheese with the sales description “Bread topping with almond produce” (E.V.A. GmbH, Oberreute, Germany), was used for the tasting as a plant-based cheese alternative (PBCA). This company is the market leader in Germany, and according to a representative Forsa survey, about 6.8 million consumers in Germany have already tried the product [38].

The plant-based meat alternative (PBMA) tasted was “Vegetarian Mühlen Salami, classic” from the manufacturer Rügenwalder Mühle (Bad Zwischenahn, Germany) with the sales description “Vegetarian product in the style of a salami based on wheat, cooked.” Rügenwalder Mühle is a traditional meat-processing company that has been very successful in the vegetable protein sector for several years selling many food products in the German market. In July 2020, their vegetarian and vegan product sales share was greater than the share of their animal-based products for the first time [39,40].

### 2.4. Sensory Evaluation

The rate-all-that-apply (RATA) questionnaire is a variant of check-all-that-apply (CATA). This method consists of asking participants to rate the intensity of the sample’s attributes. Its purpose is to increase the ability to discriminate between samples with similar sensory profiles but different intensities of certain attributes [41]. The inclusion of attribute intensity scaling improves the accuracy of descriptive profiling and leads to better product differentiation than the CATA questionnaire [42,43]. According to Ares et al. [44], the selected terms can be rated on a 5-point scale (from “slightly applicable” to “very applicable”) according to applicability. Participants were asked to leave the scale blank for terms that did not apply. In addition, the overall liking of the product was ranked here on a vertical 9-point hedonic scale, with endpoints of “dislike extremely” (1) and “like extremely” (9). A trained panel (*n* = 10) from the University of Goettingen, Germany, developed an initial list of attributes. For this purpose, the trained assessors evaluated all samples in consecutive sessions and wrote down all the attributes they could think of to describe the samples, considering all relevant modalities. The sensory attributes mentioned most frequently and for which a reference or standard definition could be identified were selected for inclusion in the RATA ballot. The final lists of terms included 14–15 terms (see Supplementary Materials Table S1). During sensory testing, the attributes appeared in a fixed order. To help participants quickly find the appropriate response, the order corresponded to the expected “dynamics of sensory perception” [43,45]: appearance, odor, flavor/taste, and texture. Participants were provided with water and unsalted crackers (P. Heumann’s Matzen, Germany) to neutralize their senses.

### 2.5. Questionnaire

Participants provided initial information on sociodemographics, including gender, age, education, and household income (Table 1). Information on height and weight allowed us to draw inferences on the body mass index (BMI, kg/m^2^) of the participants.

Participants indicated that they followed an omnivorous, flexitarian, vegetarian, or vegan diet. Omnivorous and flexitarian diets were determined by reporting their meat consumption (e.g., “I eat meat regularly,” “I deliberately eat less meat”). This was verified or adjusted by asking about the consumption frequency for meals containing meat and meat products, on a 7-point scale from “never” to “more than five times a week,” as well as the type of animal-based product consumed. Accordingly, participants who consumed meat and/or meat products 3 or more times a week were classified as omnivores. Participants who consumed meat and/or meat products but no more than 1–2 times per week were classified as flexitarians [46]. Participants who did not eat meat and/or meat products but did eat other animal-based products were assigned to the vegetarian diet (no fine-grained distinctions of lacto-ovo vegetarian, pescetarian, etc.), and participants who did not eat meat or meat products or animal-based products were assigned to the vegan diet.

For each plant-based product group (i.e., PBMA, PBCA, and PBMiA), consumption characteristics were examined in more detail: (a) the frequency that participants consumed alternative products, on a 7-point scale from “never” to “more than five times a week,” adapted from Hoek et al. [47]; (b) what food or raw material the products were based on (e.g., grains, legumes, seeds); and (c) where they were purchased (e.g., discount store, supermarket, organic food store).

Ten items were used to assess participants’ motivation for consuming plant-based alternatives. Based on earlier work [6,48,49,50,51], the items related to health, environment, animal welfare, social setting, product lifestyle, sensory appeal, and convenience. Further items were developed by discussing, adjusting, and reflecting on current motivations for food choice motives (e.g., “I am interested in the advertising and the product design”) (see Appendix A, Table A1). The item on “convenience” was surveyed for PBMAs only, against the background that many PBMA products are ready-to-heat or prepared as ingredients for further processing. Participants responded to these items on a scale from 1, “totally disagree”, to 5, “totally agree”. The scale offered a “don’t know” response option.

Seven items for PBMAs and PBCAs and six items for PBMiAs related to sustainability characteristics of the products were used to assess the participants’ objective knowledge (e.g., plant-based meat alternatives are a source of vegetable protein) (see Appendix B, Table A2). Information on PBMAs was based on research by Huber and Keller [26], Nijdam et al. [52], Mejia et al. [53], Leitzmann [54], Joshi and Kumar [55], and Klementova et al. [56]. Findings on PBCAs came from the studies of Jeewanthi and Paik [57] and Masotti et al. [58], and data for PBMiAs originated from research by Röös et al. [59] and Poore and Nemecek [60]. Participants were asked to indicate the extent to which they agreed with the items on a scale of 1, “strongly disagree”, to 5, “strongly agree”. The knowledge questionnaire included a “don’t know” response option so that participants could use this option instead of guessing [61,62]. One of the items in the questionnaire contained a negative meaning; the remaining items were worded positively. The values of the negatively worded item were recoded in the data analysis so that the highest score represented the highest possible agreement, indicating correspondingly greater knowledge. Responses were averaged for the sustainability characteristics: health, environment, ultra-processed food (UPF), additives, and consistency.

### 2.6. Data Analysis

The questionnaires were recorded using EyeQuestion^®^ software (Elst, The Netherlands), and the extensive data set could be easily linked and processed with SPSS^®^ statistical software (IBM SPSS Statistics, Version 26.0, Armonk, NY, USA) and Microsoft Excel^®^ (Microsoft Office Professional Plus, 2013). Sociodemographics, motivation, and knowledge were analyzed using simple descriptive statistics to report percentages, means, and standard deviations. Differences between dietary styles were tested using the Kruskal–Wallis rank test and chi-square for percentages and mean values. When differences existed, they were determined using Bonferroni correction two-sample test. Analysis of variance (ANOVA) was performed in SPSS^®^ for all RATA questions as well as for the hedonic question on overall liking on the 9-point scale; these were subdivided by dietary styles (i.e., omnivore, flexitarian, vegetarian, vegan). Fisher’s Least Significant Difference (LSD) multiple comparison test with α = 0.05 was used for mean comparisons. A *p*-value < 0.05 indicated statistical significance.

## 3. Results

### 3.1. Study Characteristics

The characteristics of the study sample are shown in Table 1, further classified by dietary styles. Of 159 participants, about 70% were female, and almost 54% were 18–24-years-old, thus belonging to Generation Z [63]. Although the mean age of the respondents was 30.1 years (Generation Y), there were significant differences between the nutrition styles. Omnivore participants were on average older, 33.0 years, than vegetarians at 26.4 years, and vegans at 25.8 years. The above-average education is also an indicator that this study took place at a university, so 50.0% of the omnivores indicated having completed their university study. The BMI results should provide an indicator of the participants’ health status; these data are based on self-reporting. Nutritional status is based on WHO criteria, with a BMI 18.5–24.9 indicating a normal weight. Overweight is defined as having a BMI of 25 or more, and obesity is defined as having a BMI of 30 or more. With a BMI of 22.6, this cohort was within the normal weight range. With a BMI of 23.9, the omnivores had a significantly higher BMI than the vegetarians with 22.1 and vegans with 22.0. The high percentage (11.8%) of underweight vegans should be noted.

### 3.2. Sensory Consumer Profiling and Overall Liking

The sensory profiles of the three plant-based alternative products differed in scoring before and after the information treatment in the form of the questionnaire (Table 2, Table 3 and Table 4). The attributes the participants were able to select for the products can be found in the Supplementary Materials (Table S1). The frequency of selection of sensory terms varied according to the nutrition styles. Significant differences were found between different sensory modalities for the oat drink: appearance (“beige” and “viscous”), odor (“cereal”), taste (“nutty” and “sweet”), and texture (“viscous” and “oily”); for plant-based cheese: appearance (“yellow”), odor (“cheesy,” “nutty” and “brothy” and taste (“milky” and “umami”); for plant-based salami: taste (“meaty” and “sweet”).

The oat milk data show the most significant differences between dietary groups, but they were not present in both sessions (Table 2). Thus, for the attribute “viscous appearance” and “viscous texture,” significant differences could be found in the second session; as the omnivores voted this the lowest in the first session, only tendencies can be shown for this. However, it is noticeable for this product that the attributes “bitter taste” and “sour taste” were not used by the vegans in the first and second rounds.

For the plant-based cheese, the “cheese smell” was perceived at a significantly higher rate by the flexitarians; for the “cheese taste,” there was a tendency, and this was true for both sessions (Table 3). On the other hand, the “broth odor” was rated significantly lower by the omnivores in the first sitting. In the second sitting, there was also a tendency. Analogously, the attribute “umami taste” was perceived at a significantly lower rate by the omnivores in both sittings.

In the case of the plant-based salami, there were only two significant differences; the “meat taste” was rated lowest by the omnivores in the first session, and there was also a trend in the second session for the attribute “meat smell,” which was also rated lowest by the omnivores (Table 4).

Significant differences were found in consumers’ overall liking of the products in the dietary forms and in the two sessions, except in the second round for the plant-based salami product (Table 5). In general, it is noticeable that the products were rated relatively high on the nine-point scale. The plant-based salami never received the lowest rating from the omnivores, with a mean of 5.24 and 5.52. The other products were also generally rated lower by the omnivores than by the representatives of the other diet styles. Vegans rated the oat drink and the plant-based cheese the highest.

### 3.3. Characterization of Plant-Based Product Consumption by Participants

Table 6 displays the frequency of consumption of plant-based alternative products. Regarding PBMAs, only 3.8% of participants reported never consuming such products. On average, they were consumed predominantly on a monthly basis (i.e., more than once a month but less than once a week). PBCAs were more likely to be consumed occasionally. A total of 49.1% of participants reported consuming them less than once a month or less than once a week, and 28.3% had tried these products once. PBMiAs were most frequently used along with the plant-based products in this study. They were consumed daily by about 13.8% of the participants, while 44% reported consuming them at least once a week.

There were significant differences in the consumption of plant-based alternative products between dietary styles (Table 6). Omnivore participants infrequently (i.e., less than once a month) or never ate PBMAs at a rate of 66.7%, PBCAs at 87.9%, and PBMiAs at 84.8%. Vegetarians and vegans had a significantly higher intake of alternative products compared to omnivores; vegans had a significantly higher intake than flexitarians and vegetarians. A total of 47.1% of vegans used PBMAs daily, and 58.8% consumed PBMiAs five times a week or more.

**Figure 1:**
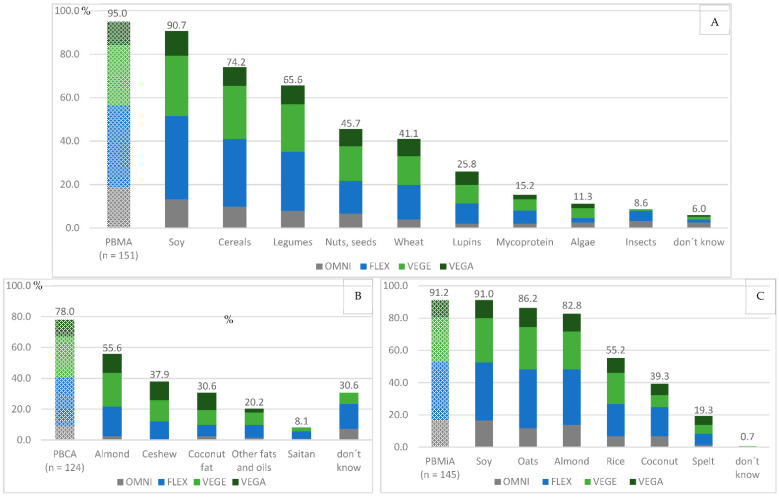
Percentage representation of the basis of the different plant-based alternative products, (**A**) = PBMA (plant-based meat alternative), (**B**) = PBCA (plant-based cheese alternative), (**C**) = PBMiA (plant-based milk alternative); patterned bars represent the total value related to the response of the participants, blank bars relate to the food or raw material basis of the products, with multiple mentions resulting in different percentages. Abbreviations of the nutrition styles: OMNI = omnivore, FLEX = flexitarian, VEGE = vegetarian, VEGA = vegan.

PBMAs with 95% (Figure 1C) and PBMiAs with 91% (Figure 1A) were the most known product groups among the respondents, so they could indicate from which raw materials these products are made. Soy, cereals, and legumes were the most mentioned sources for PBMAs, followed by nuts, seeds, and wheat (gluten). For PBMiAs, soy, oats, and almonds were the main raw material sources of products purchased and consumed by participants, at over 80%. Rice followed in fourth place with 55.2%, the product group with the lowest allergenic potential for consumers. For PBCAs, over 30.6% of consumers did not know the source of the raw materials used in the products. At about 56%, almonds were the raw materials most frequently present in the products. Figure 1B shows again that PBCAs currently still have the lowest level of awareness among consumers in the large market of plant-based alternative products.

### 3.4. Motives for Consuming Plant-Based Alternative Products

Table 7 shows that overall, for PBMAs, the strongest motives for consumption were animal welfare, followed by the environment and health. This was followed by the curiosity of the participants to try new foods and the interest aroused by design and advertising (see Appendix A, Table A1 for items). Sensory appeal and convenience were rated rather neutrally overall, with an average scale score of around 3.0, while the social environment was not really decisive for consumption. There were significant differences between the dietary styles with regard to animal welfare, the environment, and social environment. Overall, vegans and, to some extent, vegetarians, had a significantly greater agreement with the motivation items than omnivores. For vegans in particular, animal welfare was a strong motive.

With regard to PBCAs, animal welfare and the environment were also strong motives for consumption (Table 8). However, the product lifestyle was also rated with a higher agreement here. Health and sensory characteristics were rated rather neutrally, while the social environment received little approval as a motive. However, there were significant differences regarding the latter as well as with regard to animal welfare and the environment. Vegans showed significantly higher agreement than those with meat-based eating styles and, in part, also vegetarians.

Additionally, with regard to PBMiAs, the strongest motives for consumption were animal welfare and the environment (Table 9). However, sensory properties also moderately motivated consumption as well as the product design, curiosity, and advertising for PBMiAs. Health slightly motivated the consumption, while the social environment was less decisive for consumption. Differences between the dietary styles occurred in relation to animal welfare, the environment, health, and the social environment, whereby vegans and vegetarians showed a higher approval than omnivores and flexitarians.

### 3.5. Objective Knowledge of Plant-Based Alternative Products

Table 10, Table 11 and Table 12 show the participants’ objective knowledge of the sustainability aspects of plant-based alternatives. Since some of the participants answered “I do not know,” the number of responses differed from the total sample.

Participants’ overall agreement with statements about the sustainability benefits of PBMAs ranged from a value of 2.22 to 4.25. The highest level of agreement, with a value of 4.05, was related to the consistency of PBMAs, suggesting that participants viewed such products as less of a fad and more of a permanent product in the marketplace. The majority of respondents could also identify PBMAs as ultra-processed foods. There was also a higher level of agreement on environmental aspects, indicating that participants were aware that PBMAs are more environmentally friendly due to lower CO_2_ emissions than meat and meat products. Moderate agreement was found for health aspects, indicating limited knowledge among participants about health-promoting properties of PBMAs. There was low agreement on additives, to the extent that respondents did not know that organic PBMAs have fewer additives than conventionally produced PBMAs and, moreover, rated this statement as rather inaccurate. Significant differences between dietary styles occurred, particularly in relation to consistency and the environment, with vegans and vegetarians more likely to agree with these sustainability aspects than omnivores. There were also differences with respect to additives, with vegans still moderately agreeing with this characteristic (Table 10).

With regard to PBCAs, the aspect of consistency also received the highest level of agreement, meaning that participants did not rate such products as a fad, but as a product that will persist on the market. There was also agreement in terms of the degree of processing and additives, which means that respondents were largely aware of the intense processing of PBCAs as well as the supplementation of additives. Moderate knowledge was found in relation to the health aspects, with an overall score of 3.51. With regard to the health aspects, there was significantly greater agreement shown by vegetarians. No significant differences were found for additives based on the post hoc test (Table 11).

Participants’ overall agreement with statements about the sustainability benefits of PBMiAs tended to be in the upper range of agreement, with values ranging from 3.56 to 4.57. Again, most of the knowledge was related to PBMiAs’ consistency. A greater agreement, but with fewer respondents having knowledge, was found regarding the taxation of PBMiAs and cow milk. Overall, only about a third of respondents knew that plant-based milk is subject to the 19% tax rate while cows’ milk is tax-deferred as a staple food. The majority of respondents could identify PBMiAs as the more environmentally friendly food compared to cow milk. Moderate knowledge existed regarding health aspects. Significant differences between dietary styles occurred regarding the environment, with vegans agreeing significantly more with these sustainability aspects than omnivores (Table 12).

## 4. Discussion

### 4.1. Sensory Perception of Consumers

Centered sensory and consumer research can play a crucial role in enabling the optimization of sensory attributes for plant-based alternative products. Various research projects have combined qualitative and quantitative consumer studies with subsequent behavioral analyses and interviews. In this study, for example, the interviews were used as a treatment where the participants had to deal intensively with the topic of sustainable protein sources. With a high proportion of young adults as target consumers, this study showed that commercial PBCAs and PBMiAs tended to elicit higher general preferences than PBMAs.

In the study by Verbeke [64], insect-based products were able to achieve significantly higher levels of preference when consumers were informed about the products. They explained it by suggesting that young adults might be more open to trying food products prepared with insects and willing to compromise on taste when they are informed about other product benefits [51]. At this point, the initial question about consumer acceptance can be answered. The overall sensory evaluation for the plant-based products tasted here was higher after consumers were informed and treated with the questionnaire, which could not be confirmed. However, significant differences in the evaluation between the nutritional styles can be seen here. Thus, the omnivorous consumers surveyed rated the alternative products lower than vegetarians and vegans, while flexitarians rated them high at a similar rate to vegetarians (Table 5). This reflects the findings of Elzerman et al. [65], who found that meat alternative users gave higher ratings than non-users. In Table 6, it is shown that the vegetarians and vegans that were surveyed were medium-to-high users of plant-based products.

Given the low overall sensory evaluation of meat alternatives in this study, it appears that the poor sensory quality of the product may be a primary reason for the low acceptance. Since the sensory quality is an important factor in food choices, manufacturers of plant-based alternatives consider sensory attributes when developing food products. This is especially the case if they want to position their product as a meat alternative, as similarity to meat and sensory quality are crucial for consumer acceptance [47].

According to van Trijp and van Kleef [66], several factors led to the increase in acceptance of new products, such as perceived meaningfulness (usefulness to target users) and novelty (uniqueness) and the required change in existing behavior patterns. A new product should be new enough to arouse curiosity, but familiar enough not to create fear and neophobia. Product familiarity might also have played a role in the difference in evaluation. Repeated positive experiences could have led to higher acceptance by users of alternative products. This was also shown by Hoek et al. [67], who reported a pure exposure effect after repeated consumption of meat alternative products (two times per week for 10 weeks). This suggests that the more reluctant consumers’ opinion may shift toward a more positive perspective on plant-based meat products after repeated exposure to these products. Schouteten et al. [68] describe that emotions are mainly sensory-driven, as information alone has limited influence on emotional characterization. Therefore, for the market success of products with sustainable plant protein sources, it is important that potential consumers trying the product associate positive emotions with the product, replacing the expected negative emotions present before consumption. Although good taste leads to improved acceptance, familiarity plays an important role in the adoption of novel food products and should not be neglected [69].

One of the interesting findings in the present data was that the mean RATA score was very frequently above 2.5 for all dietary styles and was thus close to the scale anchor “very applicable.” This can most likely be attributed to a boundary effect. Since values higher than 5 are impossible, very consistently high values are required to reach an average score of 2.5 or higher, a consistency that is rarely observed in consumer data, according to Vidal et al. [70]. Even when an attribute is clearly present, some consumers may not select it or may assign a lower score to avoid the extreme intensity anchor on the RATA scale [70]. New insights into consumers’ ability to make detailed sensory product characterizations of plant-based oat drinks, sliced cheese, and salami style products support the results of this study. Ares et al. [41] showed that consumers performed similarly in RATA questions as trained panelists in descriptive analysis for appearance and basic taste attributes. However, consumers found it difficult to identify differences between samples in terms of complex sensory attributes or attributes related to specific flavors [41]. This may also be true for individual attributes in the results in Table 2, Table 3 and Table 4 shown here, such as astringency and umami. With brief training, it may be possible to improve consumers’ ability to describe and discriminate between complex/similar samples.

What is particularly noticeable in the RATA data on the plant-based oat milk is that vegans were the only ones in both tasting rounds who did not perceive the product as bitter or sour, and only one person found it astringent (Table 2). It has long been known that consumers have an innate preference for sweetness and an aversion to bitter-tasting foods [71]. In a recent study by Pagliarini et al. [72], a large population sample size was used to confirm the finding that the perception of sour and bitter tastes is related to eating behavior and age. Van Bussel et al.’s [73] results show that participants who eat healthier and more sustainable diets consume fewer foods from the umami, salty, fatty, and bitter taste groups than participants who eat less healthy and sustainable diets. When comparing dietary styles, this is related to lower consumption of meat products and coffee and higher consumption of fruits, vegetables, grain products, and tea [73]. Therefore, they recommend that taste profiles should be considered when suggesting healthy and sustainable menus and meals.

A larger, cross-national sample would allow further investigation into how Western consumers perceive the consumption of plant-based alternative products. In addition, the consumer tastings in this study only took place in a laboratory setting. This must be considered when interpreting the results, as the context could influence the emotional and sensory profiles of foods [74]. Therefore, future research should investigate how people evaluate this type of food in other contextual situations, such as in a restaurant, food exhibition, or supermarket.

### 4.2. Consumers’ PBAP Consumption Behavior 

The results of this study reveal that the frequency of PBAP consumption varies along with the examined products.

PBMiAs were found to be the most consumed products among those studied, with an average consumption of about one or two times per week. In fact, the market and demand for PBMiAs has grown significantly in recent years [75]. There are currently over 50 brands offering PBMiAs to German consumers, and the fact that almost all major German food retailers have entered the plant-based milk category indicates that the market for plant-based milk is already developed. According to a recent survey, 93% of German consumers already buy plant-based milk alternatives, which is higher than any of the other plant-based product categories [76]. Oat milk has recently become the most popular PBMiA ahead of soy milk [77] and is predicted to be the fastest-growing alternative in the coming years [78]. The results show that a slight majority of respondents indicated a preference for soy as a plant-based alternative milk product, albeit this was closely followed by oat milk.

PBMAs were consumed by respondents of this study more occasionally, with an average eating frequency of two to three times per month. In line with this finding, a recent consumer survey in Germany on the consumption of meat alternatives found a share of 19.3% of consumers reported consuming meat substitutes, and most of these (62.2%) were occasional users with a consumption frequency of once a month or less [20]. The use of these products in the daily diet of consumers in Germany remains low for now, as in other European countries [47,79,80,81], and a shift in consumer dietary habits to replace meat with a plant-based alternative is progressing slowly. Nevertheless, increasing concerns about the sustainability of meat production and the rise of vegetarian and vegan lifestyles have increased the demand and market for meat alternatives [10,67]. Experts predict an annual growth rate of 20% to 30% worldwide [82], and the supply in restaurants and canteens is also likely to increase. At this point, further accompanying scientific surveys are crucial to give a more accurate prediction of consumer acceptance of PBAPs in the community food supply.

Among the studied alternative products, PBCAs are the least consumed, although this result was not unexpected. Within the plant-based product market, PBCAs is a product group that has yet to gain traction and interest among a broad consumer base. Although sales of PBCAs continue to grow, such products are still in the early stages of development compared to other plant-based alternatives (i.e., milk and meat) [57,83], and consumers in supermarkets have limited options to choose from [28].

In terms of the consumption behavior of respondents following different dietary styles, the analysis indicated that PBAPs were strongly represented in vegan and vegetarian diets. On the other hand, flexitarians described lower consumption, as did omnivores. This is consistent with other studies that have found no increased consumption of meat alternatives among frequent meat eaters and moderate meat eaters who are willing to substitute meat [84,85]. Consumers who avoid meat and meat products are instead more likely to consume PBAPs, as shown by Haas et al. [86] and Ohlau et al. [87]. In this context, there is also the likelihood that the higher consumption within plant-based dietary styles correlates with sociodemographic characteristics of the sample. A higher proportion of the sample consisted of women, and the group of vegetarians and vegans was younger in age than the meat eaters. Previous studies showed that predominantly women follow a plant-based diet [86,87], and some studies showed a higher prevalence of plant-based eaters among younger age groups [88,89]. In addition, women and younger individuals were found to be more likely to consume meat substitutes [79,87].

### 4.3. Related Motives for PBAP Consumption

The reported reasons for consuming PBAPs revealed that animal welfare followed by environmental concerns were the main reasons given for consumption. Animal welfare is often cited as the primary motivation for consumers reducing or avoiding the consumption of animal products [84,90,91]. Fresán et al. [92] also found that it is the main motivation for continuing the diet. Environmental concerns are also commonly named as an important reason for consumer demand for alternative proteins [47,91,93,94]. However, Sanchez-Sabate and Sabaté [94] indicated in their review that only a small minority of consumers are willing to stop or significantly reduce meat consumption for environmental reasons or have already changed their meat intake for ecological concerns. Nevertheless, motives can differ in the strength of their influence according to dietary behavior [95]. For example, Hoek et al. [47] found that higher interest in ecological well-being is a driver of more frequent consumption of meat alternatives. Consistently, the results of the present study show that the stated sustainability-specific motivations for consuming PBAPs increased along with the strictness of the plant-based diet, such that vegans and to some extent vegetarians showed greater agreement than omnivores and to some extent flexitarians. Previous studies suggest that people who completely avoid eating animal products tend to be more ethically motivated than people who partially avoid eating animal products; this is true for vegans more so than vegetarians [96,97,98,99,100], and flexitarians more closely resemble traditional meat consumers than vegetarians and vegans in terms of their motivations related to animals and the environment [90,101,102,103]. A major reason that prevents meat eaters and flexitarians from eating a plant-based diet or PBAPs is their preference for the taste of meat [104,105].

Overall, the sensory appeal as a motive was found to be negligible to not applicable in this study. This agrees with studies that revealed that consumers perceive PBMAs as less sensory-attractive [20,47,80,106].

In terms of health, the present results show rather indifferent ratings as a consumption motive. While it seems to be somewhat more important for PBMAs, it is less crucial for the consumption of PBCAs and PBMiAs. Although numerous studies describe health concerns as one of the most important reasons for switching to a plant-based diet [92,102,107,108,109,110], it is possible that consumers may not rate PBAPs as healthy themselves. As the health assessment database for PBAPs grows, concerns are generally raised about the level of processing of plant-based substitutes [27,111] and about their partial nutrient deficiencies compared to their animal counterparts [112]. It stands to reason that while consumers may see a health benefit in a plant-based diet, they are cautious in transferring such an assessment to substitute products.

### 4.4. Consumers’ Objective Knowledge of Sustainability Characteristics of PBAPs

Although the assessment of PBAPs’ sustainability attributes is still in its infancy and needs to be expanded and verified, the analysis of participants’ objective knowledge in this study was helpful in gaining better insight into consumer perceptions of PBAPs. One intriguing finding was that respondents, regardless of the product, possessed higher agreement that PBAPs are not a fad but will be in the marketplace in the long term. In fact, market forecasts predict a significant growth of such products in the coming years [113]. Consumers’ assessment of this is critical, as it can play an important role in long-term acceptance and integration into dietary habits.

Additionally, the higher compliance to PBMAs and PBCAs as compared to ultra-processed foods was striking. Indeed, there is a current debate concerning the degree of processing of plant-based alternative products [111]. Like conventionally processed meats, some PBAPs, such as meat imitations, are highly processed and, for example, high in sodium, saturated fat, and often added sugars [29,114]. In a similar respect, studies have found that consumers perceive PBAPs as more artificial and less natural [20,115]. It is expected that the processing of PBAPs may well represent a barrier to their consumption. Additional measurement of this correlation could be useful to ultimately better predict consumer perceptions and attitudes toward PBAPs as ultra-processed foods.

The respondents had a better knowledge of the environmental benefits of PBMAs than they did for PBMiAs, which nevertheless indicates that respondents’ perceptions of the environmental friendliness of such products generally do not match the objective assessment based on environmental measurements. This further suggests that participants are not aware that plant-based alternatives are generally more environmentally friendly than meat- and animal-derived products. This result supports previous findings from Siegrist and Hartman [79] and Hartmann et al. [115] that consumers are unable to assess the environmental friendliness of products sufficiently. Hartmann et al. [115], who examined in detail whether consumers’ knowledge of the environmental friendliness of different products was consistent with life cycle assessment (LCA) data, concluded that the environmental friendliness of animal products was greatly overestimated, while meat substitutes were perceived as far less environmentally friendly than they are. Efforts should be made, for example, in the form of an environmental label, to inform and educate consumers about the environmental impact of individual products, not only PBAPs but also meat and meat products.

Respondents seemed to have lacked knowledge of certain health aspects of PBCAs and PBMiAs. For example, there was only modest agreement among respondents that, for example, with respect to cheese substitutes, such products have no lactose and no cholesterol due to the absence of animal ingredients. Overall, since the PBAP segment is still relatively new and continuously growing, it can be assumed that consumers have not (yet) gained sufficient knowledge about these products. In addition, Bucher et al. [116] found that consumers tended to neglect the number of single nutrients, such as saturated fat, protein, and sodium, in their ratings, which further leads to assuming that consumers are more likely to make general statements about health ratings rather than evaluate specific characteristics.

Lastly, it should be noted that knowledge levels partially significantly varied along with the different dietary groups, with vegans and vegetarians showing higher knowledge levels than omnivores. For example, with regard to the environmental friendliness compared to the respective animal product or the fact that organically produced PBMAs contain fewer additives than conventional PBMAs, vegans and partial vegetarians were in agreement. Thus, plant-oriented consumers may be more interested in the properties of such foods and more likely to seek knowledge than meat eaters, who consume less of such products. A study by Hartmann et al. [117] found that people with a pro-ecological orientation have more knowledge about the environmental impact of food. Similarly, Michel et al. [20] reported in their study that non-meat eaters perceive meat alternatives to be better in terms of environmental friendliness than meat eaters, although only the perception was examined here.

In general, respondents’ knowledge regarding the current sustainability aspects of PBAPs seems to be still insufficient. Studies indicate that relevant knowledge is a prerequisite to enable consumers to make environmentally friendly choices [30,117]. However, with a wide and rapidly growing range of products, there is a lot of information in the media about the valuation of PBAPs, causing a risk of consumer confusion and misinformation. For both science and consumers, the alternative product segment is still young, and more scientific evaluations need to be generated as well as general educational efforts to provide consumers with helpful and accurate information.

## 5. Limitations

The sample used was not representative of the German population due to convenience sampling during recruitment. Because of the high proportion of students, the sample consisted mainly of younger people. Furthermore, the public announcement of the sensory tasting of substitute products primarily addressed individuals with vegetarian and vegan diets. Yet, using a random sample is not unusual in sensory research. Additionally, the sample size was sufficient for the method of valid sensory testing and provided interesting findings. In terms of consumer research (i.e., consumption behavior, motivation, and knowledge), however, the treatment group might have been small. Nevertheless, this study fulfilled the standard criteria in light of sensory science, making valuable connections to the field of consumer research.

With regard to the sensory test, only three products, one from each product segment, were included in this study for practical reasons. Although market-leading products were selected, studies on a wider variety of food categories are needed to generalize the conclusions of the current study.

Further, the CATA and RATA assessments were conducted using attributes pre-sampled by a trained sensory panel. Thus, the vocabulary may have been too subject-specific for the lay consumers. It might be interesting to perform a profiling of the products with a consumer panel to gain a deeper insight into the sensory perception of the consumers towards PBAPs.

The questionnaire used to assess the respondents’ objective knowledge was designed to test laypersons’ factual knowledge of the properties of certain plant-based substitutes. We focused on sustainability aspects such as the environmental friendliness of PBMAs versus meat or degree of processing of PBMAs and PBCAs. In doing so, care was taken to use very specific information since the product range and thus the formulations of the individual products are very diverse, and general statements about generic properties, such as environmental friendliness, are difficult to make. For example, instead of stating “plant-based milk alternatives are more environmentally friendly than cows’ milk,” it stated “more land area is required to produce one liter of cows’ milk than to produce one liter of oat milk.” However, such a query might be too specific to the consumer and provide only limited results about consumer knowledge, even if it does offer interesting insights into consumer perceptions of detailed characteristics.

## 6. Conclusions

The variety of novel protein alternatives on the market is increasing, and there are many new product innovations potentially prompting consumers to change their nutrition habits. PBAPs may replace and complement meat- and animal-derived products in the human diet, potentially reducing the environmental impact of food consumption. However, there is a shared negative perception of plant-based dishes’ taste; in particular, plant-based protein products that mimic meat and animal products are associated with a reputation of negative sensory characteristics.

This sensory study showed that all PBAPs tested achieved good acceptance on average. In terms of the products used, PBMiAs and PBCAs were rated slightly better than PBMAs. However, particularly noticeable were the differences in ratings between dietary styles. Vegetarians and vegans gave a significantly higher rating to the products than omnivores and, to some extent, flexitarians.

Therefore, meat alternative products are of particular importance to meat-eating consumers because they offer a way to reduce meat consumption. If they do not like the taste, they are less likely to try these products again, despite the knowledge that switching to a more sustainable, plant-based diet has positive environmental and health impacts. Omnivores and flexitarians, who regularly consume animal-based products, have a different sensory comparison and relationship to plant-based products than vegetarians and vegans. They are the target group of these PBAPs and therefore of particular importance for further sensory research projects.

## Figures and Tables

**Table 1:** General characteristics of the study sample by nutrition style.

		All	Omnivore	Flexitarian	Vegetarian	Vegan	*p*-Value
	*n*	159	36	62	44	17	
	%		22.6	39.0	27.7	10.7	
Gender	Female	69.8	55.6	61.3	84.1	94.1	
	Male	30.2	44.4	38.7	15.9	5.9	0.002 ^1^
Age	18–24 years	53.5	27.8	50.0	75.0	64.7	
	25–29 years	22.6	30.6	24.2	11.4	29.4	
	30–39 years	12.6	22.2	12.9	9.1	0.0	
	40–70 years	11.3	19.4	12.9	4.5	5.9	0.008 ^1^
Age	Mean (SD)	30.13 (10.18)	33.03 (10.98) ^b^	32.29 (12.25) ^ab^	26.41 (4.91) ^a^	25.82 (4.77) ^a^	<0.001 ^2^
Education ^3^	Low	3.1	2.8	4.8	2.3	0.0	
	Medium	62.3	47.2	51.6	77.3	94.1	
	High	34.6	50.0	43.5	20.5	5.9	0.005 ^1^
Net household income per month	<EUR 600	37.7	36.1	29.0	40.9	64.7	
	>EUR 600–1800	37.7	19.4	46.8	45.5	23.5	
	>EUR 1800–3600	15.1	27.8	12.9	9.1	11.8	
	>EUR 3600	9.4	16.7	11.3	4.5	0.0	0.013 ^1^
Body mass index	<18.4	5.7	5.6	4.8	4.5	11.8	
	18.5–24.9	75.5	52.8	82.3	88.6	64.7	
	25.0–29.9	13.8	33.3	8.1	4.5	17.6	
	>30	5.0	8.3	4.8	2.3	5.9	0.012 ^1^
Body mass index	Mean (SD)	22.59 (3.75)	23.94 (4.31) ^b^	22.38 (3.40) ^ab^	22.12 (3.68) ^a^	21.97 (8.86) ^a^	0.009 ^2^

^a,b^ Values with the same superscript letter within the row indicate statistically nonsignificant differences between nutritional styles based on the Kruskal–Wallis rank test, and when differences existed, they were determined using the Bonferroni correction two-sample test, *p* < 0.05. ^1^ Pearson chi-square. ^2^ Kruskal–Wallis rank test. ^3^ Education classification: Low (secondary school certificate), Medium (high school graduation), High (completed studies).

**Table 2:** RATA attribute evaluation of plant-based oat drink in two sessions on a 5-point scale, with different shares of 159 participants. Significant differences could be calculated for the attributes in **bold**.

		1st Session				2nd Session			
Attribute *		Omnivore	Flexitarian	Vegetarian	Vegan	Omnivore	Flexitarian	Vegetarian	Vegan
**Beige_A**	N (% of total)	32 (20.8)	63 (40.9)	44 (28.6)	15 (9.7)	32 (20.8)	64 (41.6)	43 (27.9)	15 (9.7)
	Mean (95% CI)	4.41 (4.08–4.73) ^b^	4.21 (3.98–4.44) ^ab^	3.93 (3.64–4.22) ^a^	4.20 (3.77–4.63) ^ab^	4.28 (3.95–4.61)	4.13 (3.88–4.37)	4.23 (4.01–4.45)	4.00 (3.49–4.51)
Grey_A	N (% of total)	7 (29.2)	10 (41.7)	6 (25.0)	1 (4.2)	10 (32.3)	12 (38.7)	9 (29.0)	0
	Mean (95% CI)	2.00 (1.08–2.92)	3.20 (2.32–4.08)	3.00 (1.01–4.99)	4.00	2.50 (1.53–3.47)	2.83 (1.79–3.88)	3.00 (1.85–4.15)	
**Viscous_A**	N (% of total)	13 (17.1)	32 (42.1)	25 (32.9)	6 (7.9)	12 (15.6)	31 (40.3)	29 (37.7)	5 (6.5)
	Mean (95% CI)	3.31 (2.59–4.02)	3.41 (3.00–3.81)	3.48 (2.97–3.99)	3.83 (2.80–4.87)	2.33 (1.65–3.02) ^a^	3.26 (2.78–3.73) ^b^	3.17 (2.68–3.66) ^ab^	3.60 (2.49–4.71) ^ab^
**Cereal_O**	N (% of total)	13 (13.8)	43 (45.7)	27 (28.7)	11 (11.7)	17 (16.8)	42 (41.6)	32 (31.7)	10 (9.9)
	Mean (95% CI)	3.23 (2.44–4.02) ^a^	3.81 (3.44–4.19) ^ab^	4.00 (3.69–4.31) ^b^	3.73 (3.05–4.41) ^ab^	3.47 (2.76–4.18)	3.98 (3.66–4.30)	3.91 (3.50–4.31)	3.60 (2.76–4.44)
Milky_O	N (% of total)	11 (19.6)	25 (44.6)	19 (33.9)	1 (1.8)	10 (20.0)	23 (46.0)	14 (28.0)	3 (6.0)
	Mean (95% CI)	3.45 (2.76–4.15)	3.48 (3.07–3.89)	3.42 (2.91–3.94)	5.00	2.60 (1.83–3.37)	3.39 (2.84–3.94)	3.14 (2.51–3.78)	4.00
Nutty_O	N (% of total)	14 (19.7)	30 (42.3)	19 (26.8)	8 (11.3)	13 (19.1)	30 (44.1)	22 (32.4)	3 (4.4)
	Mean (95% CI)	3.50 (2.79–4.21)	3.43 (3.02–3.85)	3.63 (3.14–4.12)	4.13 (3.59–4.66)	3.46 (2.78–4.14)	3.10 (2.63–3.57)	3.68 (3.29–4.08)	3.33 (0.46–6.20)
Bitter_T	N (% of total)	6 (31.6)	7 (36.8)	6 (31.6)	0	4 (12.1)	15 (45.5)	14 (42.4)	0
	Mean (95% CI)	1.67 (1.12–2.21)	4.14 (3.79–4.49)	2.67 (0.95–4.38)		3.75 (1.75–5.75)	2.93 (2.22–3.64)	3.43 (2.65–4.20)	
Cereal_T	N (% of total)	27 (18.2)	62 (41.9)	44 (29.7)	15 (10.1)	29 (19.1)	64 (42.1)	42 (27.6)	17 (11.2)
	Mean (95% CI)	4.26 (3.90–4.62)	4.29 (4.07–4.51)	4.20 (3.94–4.47)	4.33 (4.06–4.60)	4.31 (3.93–4.69)	4.31 (4.10–4.53)	4.36 (4.12–4.59)	4.06 (3.67–4.44)
**Nutty_T**	N (% of total)	26 (22.4)	49 (42.2)	34 (29.3)	7 (6.0)	25 (23.4)	44 (41.1)	30 (28.0)	8 (7.5)
	Mean (95% CI)	3.27 (2.73–3.81) ^a^	3.84 (3.57–4.10) ^b^	3.88 (3.54–4.22) ^b^	3.86 (3.03–4.69) ^ab^	3.72 (3.17–4.27)	3.57 (3.24–3.90)	3.60 (3.23–3.97)	3.75 (3.16–4.34)
Sour_T	N (% of total)	2 (14.3)	5 (35.7)	7 (50.0)	0	6 (26.1)	7 (30.4)	10 (43.5)	0
	Mean (95% CI)	2.50 (1.96–2.65)	3.00 (1.24–4.76)	3.71 (2.55–4.87)		2.83 (1.44–4.23)	3.29 (2.13–4.45)	3.20 (2.09–4.31)	
**Sweet_T**	N (% of total)	26 (19.3)	54 (40.0)	39 (28.9)	16 (11.9)	27 (18.6)	61 (42.1)	40 (27.6)	17 (11.7)
	Mean (95% CI)	3.85 (3.38–4.31)	3.83 (3.60–4.07)	3.82 (3.51–4.13)	4.06 (3.57–4.56)	3.41 (2.94–3.88) ^a^	3.80 (3.56–4.05) ^ab^	3.90 (3.60–4.20) ^b^	4.24 (3.95–4.52) ^b^
Astringent_TX	N (% of total)	9 (26.5)	14 (41.2)	10 (29.4)	1 (2.9)	12 (29.3)	17 (41.5)	12 (29.3)	0
	Mean (95% CI)	2.89 (1.91–3.86)	2.93 (2.13–3.73)	3.40 (2.50–4.30)	4.00	2.67 (1.84–3.49)	2.47 (1.81–3.13)	3.50 (2.62–4.38)	
**Viscous_TX**	N (% of total)	11 (22.0)	18 (36.0)	16 (32.0)	5 (10.0)	9 (18.8)	23 (47.9)	11 (22.9)	5 (10.4)
	Mean (95% CI)	3.00 (2.40–3.60)	3.00 (2.39–3.61)	3.38 (2.73–4.02)	3.20 (1.84–4.56)	2.67 (1.90–3.44) ^a^	2.70 (2.24–3.16) ^a^	3.91 (3.44–4.38) ^b^	3.20 (1.58–4.82) ^ab^
**Oily_TX**	N (% of total)	11 (17.7)	27 (43.5)	20 (32.3)	4 (6.5)	13 (18.3)	27 (38.0)	23 (32.4)	8 (11.3)
	Mean (95% CI)	3.18 (2.24–4.12)	3.15 (2.65–3.65)	3.15 (2.69–3.61)	3.50 (0.74–6.26)	2.54 (1.73–3.34) ^a^	3.33 (2.89–3.77) ^ab^	3.61 (3.11–4.11) ^b^	3.50 (2.41–4.59) ^ab^

* _A = appearance, _O = odor, _T = taste, _TX = texture; ^a,b^ significance according to Fisher’s LSD α = 0.05.

**Table 3:** RATA attribute assessment of plant-based cheese in two sessions on a 5-point scale, with different shares of 159 participants. Significant differences could be calculated for the attributes in **bold**.

		1st Session				2nd Session			
Attribute *		Omnivore	Flexitarian	Vegetarian	Vegan	Omnivore	Flexitarian	Vegetarian	Vegan
**Yellow_A**	N (% of total)	33 (20.9)	64 (40.5)	44 (27.8)	17 (10.8)	33 (20.8)	65 (40.9)	44 (27.7)	17 (10.7)
	Mean (95% CI)	4.85 (4.72–4.98)	4.89 (4.81–4.97)	4.77 (4.64–4.90)	4.94 (4.82–5.07)	4.79 (4.64–4.94) ^ab^	4.88 (4.79–4.96) ^b^	4.77 (4.64–4.90) ^ab^	4.65 (4.39–4.90) ^a^
Glossy_A	N (% of total)	3 (18.8)	8 (50.0)	4 (25.0)	1 (6.3)	8 (32.0)	8 (32.0)	7 (28.0)	2 (8.0)
	Mean (95% CI)	3.67 (1.24–7.46)	3.38 (2.20–4.55)	3.50 (1.74–6.26)	3.00	2.13 (1.08–3.17)	2.75 (1.28–4.22)	3.14 (1.69–4.60)	4.50 (0.85–5.85)
**Cheesy_O**	N (% of total)	33 (21.6)	60 (39.2)	43 (28.1)	17 (11.1)	32 (21.2)	60 (39.7)	42 (27.8)	17 (11.3)
	Mean (95% CI)	4.27 (3.86–4.68) ^a^	4.67 (4.46–4.88) ^b^	4.51 (4.27–4.76) ^ab^	4.59 (4.33–4.85) ^ab^	4.28 (3.96–4.60) ^ab^	4.62 (4.45–4.78) ^b^	4.52 (4.31–4.73) ^ab^	4.12 (3.52–4.72) ^a^
**Nutty_O**	N (% of total)	15 (22.4)	26 (38.8)	17 (25.4)	9 (13.4)	16 (21.9)	25 (34.2)	24 (32.9)	8 (11.0)
	Mean (95% CI)	3.27 (2.50–4.03)	3.35 (2.88–3.82)	3.53 (3.04–4.01)	3.22 (2.08–4.36)	2.56 (1.89–3.24) ^a^	3.60 (3.14–4.06) ^b^	3.71 (3.27–4.15) ^b^	3.50 (2.73–4.27) ^ab^
Sour_O	N (% of total)	21 (23.1)	40 (44.0)	24 (26.4)	6 (6.6)	21 (20.8)	40 (39.6)	30 (29.7)	10 (9.9)
	Mean (95% CI)	3.43 (2.94–3.92)	3.40 (3.03–3.77)	3.25 (2.78–3.72)	3.83 (2.80–4.87)	3.19 (2.62–3.76)	3.58 (3.22–3.93)	3.17 (2.65–3.69)	3.20 (2.26–4.14)
**Brothy_O**	N (% of total)	23 (18.3)	50 (39.7)	38 (30.2)	15 (11.9)	21 (17.1)	46 (37.4)	41 (33.3)	15 (12.2)
	Mean (95% CI)	3.52 (3.02–4.02) ^a^	3.76 (3.46–4.06) ^ab^	3.84 (3.53–4.15) ^ab^	4.27 (3.88–4.66) ^b^	3.86 (3.37–4.34)	3.89 (3.51–4.27)	4.05 (3.78–4.32)	4.33 (4.06–4.60)
Cereal_O	N (% of total)	7 (25.9)	11 (40.7)	8 (29.6)	1 (3.7)	9 (26.5)	13 (38.2)	8 (23.5)	4 (11.8)
	Mean (95% CI)	3.86 (3.03–4.69)	2.73 (1.87–3.58)	3.63 (2.74–4.51)	4.00	1.89 (0.91–2.86)	2.46 (1.62–3.30)	2.88 (1.83–3.92)	2.75 (0.36–5.14)
Cheesy_T	N (% of total)	27 (19.4)	58 (41.7)	38 (27.3)	16 (11.5)	29 (19.7)	61 (41.5)	41 (27.9)	16 (10.9)
	Mean (95% CI)	4.26 (3.94–4.58)	4.33 (4.13–4.52)	4.18 (3.94–4.42)	4.25 (3.94–4.56)	4.00 (3.66–4.34)	4.33 (4.13–4.53)	4.24 (3.95–4.53)	3.94 (3.37–4.50)
**Milky_T**	N (% of total)	17 (21.5)	30 (38.0)	25 (31.6)	7 (8.9)	20 (22.2)	32 (35.6)	34 (37.8)	4 (4.4)
	Mean (95% CI)	3.65 (3.29–4.01)	3.60 (3.25–3.95)	3.60 (3.14–4.06)	3.43 (2.38–4.48)	3.10 (2.53–3.67) ^a^	3.66 (3.36–3.95) ^b^	3.56 (3.30–3.82) ^ab^	3.75 (1.75–5.75) ^ab^
Salty_T	N (% of total)	27 (18.9)	59 (41.3)	44 (30.8)	13 (9.1)	26 (17.9)	60 (41.4)	42 (29.0)	17 (11.7)
	Mean (95% CI)	4.07 (3.64–4.51)	3.93 (3.65–4.21)	4.05 (3.72–4.37)	4.00 (3.51–4.49)	4.12 (3.73–4.50)	4.08 (3.83–4.33)	3.93 (3.59–4.27)	3.88 (3.41–4.36)
Sweet_T	N (% of total)	6 (20.7)	13 (44.8)	9 (31.0)	1 (3.4)	10 (34.5)	11 (37.9)	6 (20.7)	2 (6.9)
	Mean (95% CI)	3.67 (2.81–4.52)	2.62 (1.74–3.49)	3.11 (1.99–4.23)	4.00	2.80 (1.86–3.74)	2.73 (1.99–3.47)	3.50 (2.62–4.38)	3.00 (1.71–5.17)
**Umami_T**	N (% of total)	26 (17.8)	60 (41.1)	43 (29.5)	17 (11.6)	25 (17.5)	60 (42.0)	41 (28.7)	17 (11.9)
	Mean (95% CI)	3.96 (3.56–4.37) ^a^	4.12 (3.93–4.30) ^ab^	4.26 (4.01–4.50) ^ab^	4.47 (4.15–4.79) ^b^	3.84 (3.45–4.23) ^a^	4.08 (3.85–4.31) ^ab^	4.37 (4.17–4.56) ^b^	4.29 (3.79–4.80) ^ab^
Creamy_TX	N (% of total)	30 (20.4)	63 (42.9)	37 (25.2)	17 (11.6)	27 (18.8)	61 (42.4)	40 (27.8)	16 (11.1)
	Mean (95% CI)	4.07 (3.70–4.43)	4.05 (3.78–4.31)	4.14 (3.82–4.45)	4.00 (3.55–4.45)	3.70 (3.25–4.15)	3.98 (3.73–4.24)	4.15 (3.91–4.39)	3.81 (3.16–4.46)
Juicy_TX	N (% of total)	23 (20.0)	48 (41.7)	29 (25.2)	15 (13.0)	22 (19.5)	49 (43.4)	30 (26.5)	12 (10.6)
	Mean (95% CI)	3.65 (3.17–4.13)	3.79 (3.47–4.11)	3.93 (3.59–4.27)	4.20 (3.89–4.51)	3.55 (3.00–4.09)	3.67 (3.31–4.03)	3.83 (3.51–4.16)	3.83 (3.38–4.29)
Sticky_TX	N (% of total)	14 (35.9)	16 (41.0)	7 (17.9)	2 (5.1)	14 (30.4)	19 (41.3)	9 (19.6)	4 (8.7)
	Mean (95% CI)	2.86 (2.05–3.67)	3.25 (2.54–3.96)	3.29 (2.13–4.45)	2.50 (1.56–3.15)	2.36 (1.55–3.16)	3.05 (2.49–3.62)	2.89 (1.99–3.79)	3.00 (1.16–4.84)

* _A = appearance, _O = odor, _T = taste, _TX = texture; ^a,b^ significance according to Fisher’s LSD α = 0.05.

**Table 4:** RATA attribute evaluation of plant-based salami in two sessions on a 5-point scale, with different shares of 159 participants. Significant differences could be calculated for the attributes in **bold**, without the vegan nutrition style.

		1st Session			2nd Session		
Attribute *		Omnivore	Flexitarian	Vegetarian	Omnivore	Flexitarian	Vegetarian
Red_A	N (% of total)	33 (23.6)	64 (45.7)	43 (30.7)	32 (23.0)	64 (46.0)	43 (30.9)
	Mean (95% CI)	4.36 (3.99–4.74)	4.50 (4.29–4.71)	4.53 (4.33–4.74)	4.28 (3.86–4.70)	4.42 (4.20–4.64)	4.65 (4.49–4.81)
Glossy_A	N (% of total)	13 (21.7)	29 (48.3)	18 (30.0)	13 (21.7)	26 (43.3)	21 (35.0)
	Mean (95% CI)	3.15 (2.27–4.04)	3.72 (3.37–4.07)	3.44 (2.87–4.02)	3.15 (2.24–4.07)	3.23 (2.81–3.65)	3.14 (2.64–3.65)
Brothy_O	N (% of total)	29 (21.6)	63 (47.0)	42 (31.3)	30 (22.1)	63 (46.3)	43 (31.6)
	Mean (95% CI)	4.52 (4.20–4.83)	4.65 (4.50–4.80)	4.67 (4.48–4.86)	4.40 (4.05–4.75)	4.59 (4.41–4.76)	4.53 (4.34–4.73)
Meaty_O	N (% of total)	32 (23.5)	62 (45.6)	42 (30.9)	30 (22.7)	61 (46.2)	41 (31.1)
	Mean (95% CI)	4.28 (3.94–4.63)	4.53 (4.31–4.75)	4.62 (4.42–4.81)	4.07 (3.66–4.47)	4.26 (4.03–4.49)	4.46 (4.25–4.68)
Cereal_O	N (% of total)	6 (27.3)	8 (36.4)	8 (36.4)	6 (33.3)	8 (44.4)	4 (22.2)
	Mean (95% CI)	2.17 (0.94–3.39)	3.00 (1.66–4.34)	2.88 (1.36–4.39)	2.67 (1.09–4.25)	1.88 (1.34–2.41)	3.25 (0.86–5.64)
Paprika_O	N (% of total)	17 (21.5)	36 (45.6)	26 (32.9)	19 (24.4)	35 (44.9)	24 (30.8)
	Mean (95% CI)	2.88 (2.16–3.61)	3.19 (2.75–3.64)	3.15 (2.72–3.59)	3.47 (2.91–4.04)	3.57 (3.24–3.91)	3.38 (2.85–3.90)
**Meaty_T**	N (% of total)	25 (21.4)	56 (47.9)	36 (30.8)	25 (20.5)	57 (46.7)	40 (32.8)
	Mean (95% CI)	3.44 (2.92–3.96) ^a^	3.89 (3.60–4.18) ^ab^	4.22 (3.94–4.50) ^b^	3.84 (3.40–4.28)	3.86 (3.59–4.13)	4.13 (3.83–4.42)
Cereal_T	N (% of total)	9 (25.7)	16 (45.7)	10 (28.6)	9 (31.0)	13 (44.8)	7 (24.1)
	Mean (95% CI)	3.22 (2.22–4.22)	2.75 (2.04–3.46)	2.80 (1.69–3.91)	2.78 (1.94–3.62)	2.69 (1.90–3.49)	3.29 (2.01–4.56)
Pepper_T	N (% of total)	23 (19.8)	55 (47.4)	38 (32.8)	22 (18.3)	56 (46.7)	42 (35.0)
	Mean (95% CI)	3.83 (3.36–4.29)	3.87 (3.58–4.16)	4.08 (3.79–4.37)	3.41 (2.89–3.93)	3.91 (3.67–4.15)	3.88 (3.53–4.23)
Salty_T	N (% of total)	27 (20.8)	61 (36.9)	42 (32.3)	26 (20.3)	61 (47.7)	41 (32.0)
	Mean (95% CI)	3.96 (3.58–4.35)	3.95 (3.71–4.19)	4.21 (3.96–4.47)	3.92 (3.50–4.35)	4.15 (3.94–4.35)	4.12 (3.88–4.37)
**Sweet_T**	N (% of total)	8 (28.6)	12 (42.9)	8 (28.6)	7 (26.9)	15 (57.7)	4 (15.4)
	Mean (95% CI)	3.13 (2.08–4.17)	2.83 (1.90–3.77)	2.75 (1.35–4.15)	3.71 (3.02–4.41) ^b^	2.53 (1.78–3.28) ^a^	2.50 (0.45–4.55) ^ab^
Umami_T	N (% of total)	25 (19.7)	61 (48.0)	41 (32.3)	27 (21.4)	57 (45.2)	42 (33.3)
	Mean (95% CI)	4.16 (3.70–4.62)	4.28 (4.02–4.54)	4.29 (4.05–4.54)	4.22 (3.87–4.57)	4.21 (3.97–4.45)	4.21 (3.98–4.45)
Firm_TX	N (% of total)	16 (17.6)	44 (48.4)	31 (34.1)	20 (20.0)	49 (49.0)	31 (31.0)
	Mean (95% CI)	3.56 (2.89–4.24)	3.34 (3.00–3.68)	3.58 (3.24–3.92)	3.65 (3.02–4.28)	3.29 (2.94–3.63)	3.39 (2.95–3.83)
Juicy_TX	N (% of total)	23 (21.5)	51 (47.7)	33 (30.8)	22 (20.8)	49 (46.2)	35 (33.0)
	Mean (95% CI)	3.52 (3.09–3.95)	3.63 (3.31–3.95)	3.48 (3.14–3.83)	3.55 (3.06–4.03)	3.43 (3.11–3.74)	3.57 (3.22–3.93)
Gummy_TX	N (% of total)	22 (26.5)	37 (44.6)	24 (28.9)	20 (28.2)	29 (40.8)	22 (31.0)
	Mean (95% CI)	3.68 (3.16–4.20)	3.19 (2.79–3.59)	3.17 (2.55–3.79)	3.05 (2.43–3.67)	3.28 (2.78–3.77)	3.18 (2.59–3.77)

* _A = appearance, _O = odor, _T = taste, _TX = texture; ^a,b^ significance according to Fisher’s LSD α = 0.05.

**Table 5:** Attribute overall liking for the plant-based alternative products in two sessions. Mean values of the 9-point hedonic scale with 95% confidence intervals and significance variances.

Nutrition Style	Attribute “Likes Very Much” and “Likes Extremely”	N	Oat Drink Mean (95% CI) and Significance		Cheese Mean (95% CI) and Significance		Salami Mean (95% CI) and Significance	
			1st	2nd	1st	2nd	1st	2nd
Omnivore		36	6.48 (5.92–7.05) ^a^	6.45 (5.91–7.00) ^a^	6.58 (5.90–7.25) ^a^	6.73 (6.14–7.31) ^a^	5.24 (4.42–6.06) ^a^	5.52 (4.70–6.33) ^ns^
	% within nutrition style		33.3	27.2	42.4	27.3	18.2	21.2
Flexitarian		62	7.05 (6.71–7.39) ^a^	7.11 (6.73–7.48) ^ab^	6.97 (6.58–7.35) ^a^	6.88 (6.42–7.33) ^a^	6.40 (5.93–6.87) ^a^	6.26 (5.77–6.75) ^ns^
	% within nutrition style		41.5	47.7	44.6	46.1	30.8	26.2
Vegetarian		44	7.27 (6.96–7.59) ^a^	7.02 (6.62–7.42) ^ab^	6.70 (6.10–7.31) ^a^	6.70 (6.06–7.35) ^a^	6.27 (5.68–6.86) ^b^	6.32 (5.78–6.86) ^ns^
	% within nutrition style		52.3	40.9	45.4	47.7	31.8	22.7
Vegan		17	8.06 (7.77–8.34) ^b^	7.82 (7.37–8.28) ^b^	8.18 (7.85–8.50) ^b^	8.06 (7.72–8.40) ^b^		
	% within nutrition style		88.2	76.4	88.2	82.3		

^a,b^ Significance according to Fisher’s LSD α = 0.05, ^ns^ not significant.

**Table 6:** Frequency of consumption (%) of PBMAs (plant-based meat alternatives), PBCAs (plant-based cheese alternatives), and PBMiAs (plant-based milk alternatives) of all participants and by diet.

		All n = 159	Omnivore *n* = 36	Flexitarian *n* = 62	Vegetarian *n* = 44	Vegan *n* = 17	*p*-Value
PBMAs	never	3.8	6.1	6.2	0.0	0.0	
	<1x per month	31.4	60.6	33.8	13.6	11.8	<0.001 ^1^
	>1x per month, <1x per week	27.0	21.2	27.7	34.1	17.6	
	1–2x per week	14.5	12.1	15.4	18.2	5.9	
	3–4x per week	9.4	0.0	7.7	18.2	11.8	
	≥5x per week	5.0	0.0	4.6	9.1	5.9	
	daily	8.8	0.0	4.6	6.8	47.1	<0.001 ^1^
PBMAs ^3^	Mean (SD)	3.45 (1.63)	2.39 (0.79) ^a^	3.17 (1.49) ^a^	3.95 (1.47) ^b^	5.24 (1.99) ^c^	<0.001 ^2^
PBCAs	never	22.0	51.5	24.6	4.5	0.0	<0.001 ^1^
	<1x per month	49.1	36.4	55.4	59.1	23.5	0.025 ^1^
	>1x per month, <1x per week	12.6	9.1	9.2	18.2	17.6	
	1–2x per week	7.5	3.0	4.6	4.5	35.3	<0.001 ^1^
	3–4x per week	4.4	0.0	4.6	4.5	11.8	
	≥5x per week	1.3	0.0	0.0	2.3	5.9	
	daily	3.1	0.0	1.5	6.8	5.9	
PBCAs ^3^	Mean (SD)	2.40 (1.37)	1.64 (0.78) ^a^	2.15 (1.15) ^ab^	2.80 (1.52) ^b^	3.76 (1.44) ^c^	<0.001 ^2^
PBMiAs	never	8.8	21.2	10.8	0.0	0.0	0.006 ^1^
	<1x per month	29.6	42.4	30.8	27.3	5.9	
	>1x per month, <1x per week	17.6	12.1	21.5	20.5	5.9	
	1–2x per week	10.1	12.1	9.2	9.1	11.8	
	3–4x per week	11.3	6.1	6.2	20.5	17.6	
	≥5x per week	8.8	3.0	7.7	9.1	23.5	
	daily	13.8	3.0	13.8	13.6	35.3	0.020 ^1^
PBMiAs ^3^	Mean (SD)	3.67 (1.94)	2.61 (1.52) ^a^	3.48 (1.95) ^ab^	4.05 (1.78) ^b^	5.53 (1.55) ^c^	<0.001 ^2^

^a–c^ Values with the same superscript letter indicate statistically significant differences between nutritional styles based on the Kruskal–Wallis rank test, and when differences existed, they were determined using the Bonferroni correction two-sample test, *p* < 0.05. ^1^ Pearson chi-square. ^2^ Kruskal–Wallis rank test. ^3^ Measured on a 7-point scale from “never” (1) to “daily” (7).

**Table 7:** Motives for purchasing PBMAs ^1^ by diet measured on a 5-point-scale (1 “totally disagree”, 5 “totally agree”).

	Total		Omnivore		Flexitarian		Vegetarian		Vegan		
	N	Mean (SD)	N	Mean (SD)	N	Mean (SD)	N	Mean (SD)	N	Mean (SD)	*p*-Value
Animal Welfare	148	4.41 (1.00)	30	4.00 (1.26) ^a^	57	4.37 (0.94) ^ab^	44	4.50 (0.95) ^ab^	17	5.00 (-) ^b^	0.008
Environment	145	3.78 (1.18)	28	2.93 (1.33) ^a^	56	3.91 (0.98) ^b^	44	4.02 (1.07) ^b^	17	4.12 (1.22) ^b^	<0.001
Health	149	3.63 (1.00)	30	3.33 (1.03)	58	3.72 (1.01)	44	3.66 (0.99)	17	3.76 (0.97)	0.325
Product Lifestyle	149	3.46 (0.92)	30	3.43 (0.90)	58	3.34 (0.98)	44	3.52 (0.79)	17	3.71 (1.05)	0.506
Convenience	145	3.05 (1.19)	28	2.39 (1.31) ^a^	58	3.07 (1.30) ^ab^	44	3.25 (0.97) ^b^	17	3.18 (0.88) ^ab^	0.020
Sensory appeal	147	3.01 (1.20)	28	2.43 (1.29) ^a^	56	3.04 (1.18) ^ab^	44	3.25 (1.10) ^b^	17	3.59 (0.94) ^b^	0.005
Social setting	146	2.35 (1.54)	30	2.03 (1.63)	57	2.26 (1.43)	42	2.33 (1.48)	17	3.24 (1.68)	0.068

^1^ plant-based meat alternative, ^a,b^ different subscripts indicate that the mean scores were different between the diet groups (Bonferroni test, *p* < 0.05). Deviation in N due to the answer option “I don’t know”.

**Table 8:** Motives for purchasing PBCAs ^1^ by diet measured on a 5-point-scale (1 “totally disagree”, 5 “totally agree”).

	Total		Omnivore		Flexitarian		Vegetarian		Vegan		
	N	Mean (SD)	N	Mean (SD)	N	Mean (SD)	N	Mean (SD)	N	Mean (SD)	*p*-Value
Animal Welfare	122	4.30 (1.13)	16	3.69 (1.66) ^a^	47	4.06 (1.13) ^a^	42	4.52 (0.92) ^ab^	17	5.00 (-) ^b^	0.001
Environment	122	4.11 (1.10)	16	3.56 (1.59)	47	4.21 (1.00)	42	4.12 (0.99)	17	4.29 (0.99)	0.181
Product Lifestyle	122	3.39 (0.91)	16	3.56 (0.98)	47	3.28 (0.93)	42	3.31 (0.84)	17	3.71 (1.05)	0.304
Health	121	3.01 (0.95)	15	2.80 (1.21) ^ab^	47	2.79 (0.83) ^a^	42	3.12 (0.86) ^ab^	17	3.53 (1.07) ^b^	0.029
Sensory appeal	122	2.97 (1.27)	16	2.31 (1.08) ^a^	47	2.89 (1.37) ^a^	42	2.90 (1.14) ^a^	17	3.94 (0.90) ^b^	0.002
Social setting	122	1.67 (1.26)	16	1.56 (1.37) ^a^	47	1.49 (1.08) ^a^	42	1.50 (1.02) ^a^	17	2.71 (1.69) ^b^	0.003

^1^ plant-based cheese alternative, ^a,b^ different subscripts indicate that the mean scores were different between the diet groups (Bonferroni test, *p* < 0.05). Deviation in N due to the answer option “I don’t know”.

**Table 9:** Motives for purchasing PBMiAs ^1^ by diet measured on a 5-point-scale (1 “totally disagree”, 5 “totally agree”).

	Total		Omnivore		Flexitarian		Vegetarian		Vegan		
	N	Mean (SD)	N	Mean (SD)	N	Mean (SD)	N	Mean (SD)	N	Mean (SD)	*p*-Value
Animal Welfare	143	4.31 (1.08)	26	4.00 (1.36) ^a^	56	4.13 (1.13) ^a^	44	4.50 (0.90) ^ab^	17	4.94 (0.24) ^b^	0.010
Environment	143	4.18 (1.11)	26	3.50 (1.53) ^a^	56	4.05 (1.03) ^ab^	44	4.57 (0.73) ^b^	17	4.65 (0.79) ^b^	<0.001
Sensory appeal	143	3.66 (0.29)	26	3.35 (1.47)	56	3.71 (1.32)	44	3.52 (1.19)	17	4.35 (1.00)	0.070
Product Lifestyle	143	3.64 (0.92)	26	3.73 (1.00)	56	3.50 (0.98)	44	3.77 (0.83)	17	3.65 (1.11)	0.488
Health	143	3.36 (1.15)	26	3.00 (1.30) ^a^	57	2.98 (1.13) ^a^	43	3.77 (0.92) ^b^	17	4.12 (0.86) ^b^	<0.001
Social setting	143	2.08 (1.50)	26	2.23 (1.77) ^ab^	56	1.80 (1.27) ^a^	44	1.95 (1.28) ^ab^	17	3.06 (1.92) ^b^	0.019

^1^ plant-based milk alternative, ^a,b^ different subscripts indicate that the mean scores were different between the diet groups (Bonferroni test, *p* < 0.05). Deviation in N due to the answer option “I don’t know”.

**Table 10:** Mean scores and standard deviation of the objective knowledge about sustainability characteristics of PBMAs ^1^ by participants’ diets.

	Total		Omnivore		Flexitarian		Vegetarian		Vegan		
	N	Mean	N	Mean	N	Mean	N	Mean	N	Mean	*p*-Value
Consistency	151	4.25 (0.87)	30	3.83 (1.09) ^a^	60	4.22 (0.85) ^ab^	44	4.41 (0.76) ^b^	17	4.71 (0.47) ^b^	0.004
UPF	142	4.05 (0.88)	30	4.00 (1.08)	54	4.07 (0.82)	41	4.02 (0.82)	17	4.12 (0.86)	0.966
Environment	145	3.89 (1.04)	25	3.28 (1.14) ^a^	59	3.90 (1.05) ^ab^	44	4.07 (0.87) ^b^	17	4.29 (0.92) ^b^	0.005
Additives	111	2.22 (1.11)	23	1.87 (1.06) ^a^	45	2.24 (1.21) ^ab^	33	2.15 (0.83) ^ab^	10	3.10 (1.20) ^b^	0.030

^1^ plant-based meat alternative, ^a,b^ different subscripts indicate that the mean scores were different between the diet groups (Bonferroni test, *p* < 0.05); UPF = ultra-processed food. Deviation in N due to the answer option “I don’t know”.

**Table 11:** Mean scores and standard deviation of the objective knowledge about sustainability characteristics of PBCAs ^1^ by participants’ diets.

	Total		Omnivore		Flexitarian		Vegetarian		Vegan		
	N	Mean	N	Mean	N	Mean	N	Mean	N	Mean	*p*-Value
Consistency	121	4.33 (0.82)	15	4.00 (1.25)	48	4.21 (0.71)	42	4.45 (0.80)	16	4.69 (0.48)	0.057
UPF	113	4.11 (0.90)	15	4.13 (1.25)	44	4.18 (0.79)	38	4.00 (0.84)	16	4.13 (1.03)	0.839
Additives	103	4.11 (0.78)	16	4.31 (1.01)	41	4.15 (0.65)	33	3.82 (0.73)	13	4.46 (0.78)	0.035
Health	124	3.51 (1.16)	16	3.63 (1.18) ^ab^	49	3.57 (1.17) ^ab^	42	3.15 (1.22) ^a^	17	4.07 (0.58) ^b^	0.038

^1^ plant-based cheese alternative, ^a,b^ different subscripts indicate that the mean scores were different between the diet groups (Bonferroni test, *p* < 0.05); UPF = ultra-processed food. Deviation in N due to the answer option “I don’t know”.

**Table 12:** Mean scores and standard deviation of the objective knowledge about sustainability characteristics of PBMiAs ^1^ by participants’ diets.

	Total		Omnivore		Flexitarian		Vegetarian		Vegan		
	N	Mean	N	Mean	N	Mean	N	Mean	N	Mean	*p*-Value
Consistency	142	4.57 (0.74)	25	4.32 (0.99)	57	4.56 (0.68)	44	4.61 (0.72)	16	4.88 (0.34)	0.123
VAT	49	4.27 (1.08)	4	3.25 (1.71)	19	4.42 (0.84)	17	4.35(1.00)	9	4.22 (1.30)	0.256
Environment	133	4.20 (0.91)	22	3.75 (1.11) ^a^	52	4.14 (0.83) ^ab^	43	4.31 (0.90) ^ab^	16	4.72 (0.58) ^b^	0.008
Health	104	3.56 (1.05)	13	3.42 (1.41)	45	3.60 (1.04)	33	3.42 (0.98)	13	3.92 (0.89)	0.502

^1^ plant-based milk alternative, ^a,b^ different subscripts indicate that the mean scores were different between the diet groups (Bonferroni test, *p* < 0.05); VAT = value-added tax. Deviation in N due to the answer option “I don’t know”.

## Data Availability

Data is contained within the article and supplementary material.

## Supplementary Materials

The following supporting information can be downloaded at: https://www.mdpi.com/article/10.3390/foods11152339/s1. Table S1: Sensory Attributes Survey.

## Appendix A

**Table A1:** Constructs of motivation with associated questionnaire items and sources.

Construct	Scale/Items	Source
Animal welfare	I think animal welfare is important	[6]
Environment	It’s better for the environment	[6]
Sensory appeal	I like the sensory aspects such as taste and mouthfeel	[50]
Health	I would like a plant-based alternative source of protein	[50]
	It contributes to a healthy diet for me	[48]
	It’s healthier, not frequently eating meat	[6]
Product lifestyle	I am interested in the advertising and the product design	[49]
	I am curious about new foods	[49,51]
Social setting	Others in the household don’t want to eat animal-based products	[6]
Convenience	It is easy to prepare	[48,51]

## Appendix B

**Table A2:** Questionnaire items for knowledge of the respective products and sources.

Product	Scale/Items	Source
PBMA	Plant-based meat alternatives are a source of vegetable protein	[26]
	Plant-based meat alternatives have a better fatty acid profile than meat and meat products	[26]
	Plant-based meat alternatives have lower CO_2_ emissions compared to meat and meat products	[52,53]
	Organically produced meat substitutes have fewer additives than conventionally produced meat substitutes	[26]
	Plant-based meat alternatives have equivalent amounts of salt as meat and meat products	[26]
	Plant-based meat alternatives are ultra-processed foods	[54,82,118]
	Plant-based meat alternatives are a fad and will be gone in a few years	
PBCA	Plant-based cheese alternatives are free from lactose	[57,58]
	Plant-based cheese alternatives are free of cholesterol	
	Plant-based cheese alternatives are reduced in saturated fatty acids	[58]
	Plant-based cheese alternatives have a reduced salt content	[58]
	Plant-based cheese alternatives can be fortified with important vitamins and minerals	[58]
	Plant-based cheese alternatives are ultra-processed foods	[111]
	Plant-based cheese alternatives are a fad and will be gone in a few years	[118,119,120]
PBMiA	Plant-based milk alternatives have lower calorie content compared to whole cows’ milk	[59]
	Plant-based milk alternatives have a better fatty acid profile compared to cows’ milk	[59]
	More land area is required to produce one liter of cows’ milk compared to plant-based milk alternatives.	[60]
	Plant-based milk alternatives produce fewer CO_2_ emissions.	[60]
	Cows’ milk falls under the reduced VAT rate of 7%, while plant-based milk alternatives are subject to the regular rate of 19%.	[121]
	Plant-based milk alternatives are a fad and will disappear in a few years	[78,121]
