# Supplementary material for: Plant-Based Only: Investigating Consumers’ Sensory Perception, Motivation, and Knowledge of Different Plant-Based Alternative Products on the Market
Source: Foods. 2022 Aug 5;11(15):2339. doi: 10.3390/foods11152339 (PMC9368216; doi:10.3390/foods11152339)
Supplement: Supplementary file 1 [file foods-11-02339-s001.zip › foods-1825068-supplementary.pdf]

S1: Supplement: Sensory Attributes Survey

Product 1
Select all attributes from the list below that characterize the sample at hand.
If the attribute does not apply to the sample at hand, please tick **NO.**
If the attribute applies to the sample at hand, please check **YES** and rate on the scale how much it applies.

Please take a good look at the sample first before you evaluate this.

**Appearance: beige color**	○ No	○ Yes		
○ Very little applicable	○ Hardly applicable	○ Neither	○ Slightly applicable	○ Very applicable
**Appearance: grey color**	○ No	○ Yes		
○ Very little applicable	○ Hardly applicable	○ Neither	○ Slightly applicable	○ Very applicable
**Appearance: viscous**	○ No	○ Yes		
○ Very little applicable	○ Hardly applicable	○ Neither	○ Slightly applicable	○ Very applicable

Now please hold the sample about 2cm under your nose and sniff it at least 3 times

**Odor: cereal**	○ No	○ Yes		
○ Very little applicable	○ Hardly applicable	○ Neither	○ Slightly applicable	○ Very applicable
**Odor: milky**	○ No	○ Yes		
○ Very little applicable	○ Hardly applicable	○ Neither	○ Slightly applicable	○ Very applicable
**Odor: nutty**	○ No	○ Yes		
○ Very little applicable	○ Hardly applicable	○ Neither	○ Slightly applicable	○ Very applicable

Please taste now the present sample and evaluate the following attributes.

**Flavor: bitter**	○ No	○ Yes		
○ Very little applicable	○ Hardly applicable	○ Neither	○ Slightly applicable	○ Very applicable
**Flavor: cereal**	○ No	○ Yes		
○ Very little applicable	○ Hardly applicable	○ Neither	○ Slightly applicable	○ Very applicable
**Flavor: nutty**	○ No	○ Yes		
○ Very little applicable	○ Hardly applicable	○ Neither	○ Slightly applicable	○ Very applicable
**Flavor: sour**	○ No	○ Yes		
○ Very little applicable	○ Hardly applicable	○ Neither	○ Slightly applicable	○ Very applicable
**Flavor: sweet**	○ No	○ Yes		
○ Very little applicable	○ Hardly applicable	○ Neither	○ Slightly applicable	○ Very applicable
**Texture/mouthfeel: astringent (dry contraction, harsh)**	○ No	○ Yes		
○ Very little applicable	○ Hardly applicable	○ Neither	○ Slightly applicable	○ Very applicable
**Texture/mouthfeel: viscous**	○ No	○ Yes		
○ Very little applicable	○ Hardly applicable	○ Neither	○ Slightly applicable	○ Very applicable
**Texture/mouthfeel: oily**	○ No	○ Yes		
○ Very little applicable	○ Hardly applicable	○ Neither	○ Slightly applicable	○ Very applicable

Now evaluate the overall liking for the sample

Dislike extremely	Dislike very much	Dislike moderately	Dislike slightly	Neither Like nor Dislike	Like slightly	Like moderately	Like very much	Like extremely
○	○	○	○	○	○	○	○	○

Please neutralize your senses with **Matzen® and water!**

I can not taste the sample or only partially because:

Product 2
Select all attributes from the list below that characterize the sample at hand.
If the attribute does <u>not</u> apply to the sample at hand, please tick **NO.**
If the attribute applies to the sample at hand, please check **YES** and rate on the scale how much it applies.

Please take a good look at the sample first before you evaluate this.

**Appearance: yellow color**	o No	o Yes		
o Very little applicable	o Hardly applicable	o Neither	o Slightly applicable	o Very applicable
**Appearance: glossy**	o No	o Yes		
o Very little applicable	o Hardly applicable	o Neither	o Slightly applicable	o Very applicable

Now please hold the sample about 2cm under your nose and sniff it at least 3 times

**Odor: cheesy (typical for Gouda)**	o No	o Yes		
o Very little applicable	o Hardly applicable	o Neither	o Slightly applicable	o Very applicable
**Odor: nutty**	o No	o Yes		
o Very little applicable	o Hardly applicable	o Neither	o Slightly applicable	o Very applicable
**Odor: sourly**	o No	o Yes		
o Very little applicable	o Hardly applicable	o Neither	o Slightly applicable	o Very applicable
**Odor: brothy/spicy**	o No	o Yes		
o Very little applicable	o Hardly applicable	o Neither	o Slightly applicable	o Very applicable

Please taste now the present sample and evaluate the following attributes.

**Flavor: cereal**	o No	o Yes		
o Very little applicable	o Hardly applicable	o Neither	o Slightly applicable	o Very applicable
**Flavor: cheesy (typical for Gouda)**	o No	o Yes		
o Very little applicable	o Hardly applicable	o Neither	o Slightly applicable	o Very applicable
**Flavor: milky**	o No	o Yes		
o Very little applicable	o Hardly applicable	o Neither	o Slightly applicable	o Very applicable
**Flavor: salty**	o No	o Yes		
o Very little applicable	o Hardly applicable	o Neither	o Slightly applicable	o Very applicable
**Flavor: sweet**	o No	o Yes		
o Very little applicable	o Hardly applicable	o Neither	o Slightly applicable	o Very applicable
**Flavor: umami (savory, hearty)**	o No	o Yes		
o Very little applicable	o Hardly applicable	o Neither	o Slightly applicable	o Very applicable
**Texture/mouthfeel: creamy, soft**	o No	o Yes		
o Very little applicable	o Hardly applicable	o Neither	o Slightly applicable	o Very applicable
**Texture/mouthfeel: juicy, moist**	o No	o Yes		
o Very little applicable	o Hardly applicable	o Neither	o Slightly applicable	o Very applicable

Texture/mouthfeel: sticky ○ No ○ Yes

○ Very little applicable ○ Hardly applicable ○ Neither ○ Slightly applicable ○ Very applicable

Now evaluate the overall liking for the sample

Dislike extremely	Dislike very much	Dislike moderately	Dislike slightly	Neither Like nor Dislike	Like slightly	Like moderately	Like very much	Like extremely
○	○	○	○	○	○	○	○	○

Please neutralize your senses with Matzen® and water!

I can not taste the sample or only partially because:

Product 3
Select all attributes from the list below that characterize the sample at hand.
If the attribute does <u>not</u> apply to the sample at hand, please tick **NO.**
If the attribute applies to the sample at hand, please check **YES** and rate on the scale how much it applies.

Please take a good look at the sample first before you evaluate this.

Appearance: red color ○ No ○ Yes

○ Very little applicable ○ Hardly applicable ○ Neither ○ Slightly applicable ○ Very applicable

Appearance: glossy ○ No ○ Yes

○ Very little applicable ○ Hardly applicable ○ Neither ○ Slightly applicable ○ Very applicable

Now please hold the sample about 2cm under your nose and sniff it at least 3 times

Odor: brothy/spicy ○ No ○ Yes

○ Very little applicable ○ Hardly applicable ○ Neither ○ Slightly applicable ○ Very applicable

Odor: meaty ○ No ○ Yes

○ Very little applicable ○ Hardly applicable ○ Neither ○ Slightly applicable ○ Very applicable

Odor: cereal ○ No ○ Yes

○ Very little applicable ○ Hardly applicable ○ Neither ○ Slightly applicable ○ Very applicable

Odor: paprika ○ No ○ Yes

○ Very little applicable ○ Hardly applicable ○ Neither ○ Slightly applicable ○ Very applicable

Please taste now the present sample and evaluate the following attributes.

Flavor: meaty ○ No ○ Yes

○ Very little applicable ○ Hardly applicable ○ Neither ○ Slightly applicable ○ Very applicable

Flavor: cereal ○ No ○ Yes

○ Very little applicable ○ Hardly applicable ○ Neither ○ Slightly applicable ○ Very applicable

Flavor: pepper ○ No ○ Yes

○ Very little applicable ○ Hardly applicable ○ Neither ○ Slightly applicable ○ Very applicable

Flavor: salty ○ No ○ Yes

○ Very little applicable ○ Hardly applicable ○ Neither ○ Slightly applicable ○ Very applicable

Flavor: sweet ○ No ○ Yes

○ Very little applicable ○ Hardly applicable ○ Neither ○ Slightly applicable ○ Very applicable

Flavor: umami (savory, hearty) ○ No ○ Yes

o Very little applicable	o Hardly applicable	o Neither	o Slightly applicable	o Very applicable
**Texture/mouthfeel: firm**	o No	o Yes		
o Very little applicable	o Hardly applicable	o Neither	o Slightly applicable	o Very applicable
**Texture/mouthfeel: juicy, moist**	o No	o Yes		
o Very little applicable	o Hardly applicable	o Neither	o Slightly applicable	o Very applicable
**Texture/mouthfeel: gummy**	o No	o Yes		
o Very little applicable	o Hardly applicable	o Neither	o Slightly applicable	o Very applicable

Now evaluate the overall liking for the sample

Dislike extremely	Dislike very much	Dislike moderately	Dislike slightly	Neither Like nor Dislike	Like slightly	Like moderately	Like very much	Like extremely
o	o	o	o	o	o	o	o	o

Please neutralize your senses with Matzen® and water!

I can not taste the sample or only partially because:

The sensory questions in the first part are identical to those in the second part, so they are not shown again here.

General note: The questionnaire is in the German language and is translated here.
